# Experiment-Driven Gaussian Process Surrogate Modeling and Bayesian Optimization for Multi-Objective Injection Molding

**DOI:** 10.3390/polym18080902

**Published:** 2026-04-08

**Authors:** Hanafy M. Omar, Saad M. S. Mukras

**Affiliations:** Department of Mechanical Engineering, College of Engineering, Qassim University, Buraydah 52571, Saudi Arabia; hanafy@qu.edu.sa

**Keywords:** Bayesian, optimization, Gaussian regression, high-density polyethylene, injection molding, machine learning, shrinkage, warpage

## Abstract

Injection molding process optimization has predominantly relied on simulation-generated data, which cannot capture machine-specific variability and stochastic process noise inherent in real manufacturing environments. This paper presents an experiment-driven machine learning framework for multi-objective optimization of injection molding process parameters targeting volumetric shrinkage, warpage, cycle time, and part weight. Physical experiments were conducted on an industrial injection molding machine using high-density polyethylene with a face-centered central composite design. Systematic benchmarking of four machine learning algorithms under identical cross-validation protocols identified Gaussian process regression as the best-performing surrogate model for the majority of quality metrics, while warpage prediction remained challenging across all algorithms due to its complex thermo-mechanical origins. Permutation-based feature importance analysis established a clear parameter hierarchy, identifying holding time as the dominant factor governing multiple quality responses. Constrained Bayesian optimization with progressive constraint tightening was employed to identify optimal parameter sets and fundamental process capability boundaries. The resulting parameter configurations were validated against a held-out test set. This work demonstrates that rigorous, data-driven optimization using exclusively experimental data provides a viable and practically achievable alternative to simulation-based approaches, contributing to experiment-centric smart manufacturing in polymer processing.

## 1. Introduction

Injection molding is one of the most widely adopted polymer processing technologies for mass production of plastic components, with approximately one-third of all processed polymers manufactured through this method [1,2]. The process involves a sequence of precisely controlled stages—plasticization, injection, packing, cooling, and ejection—that must be executed with careful parameter selection to ensure acceptable product quality [3,4]. Product quality is typically evaluated through four primary metrics: volumetric shrinkage, warpage, cycle time, and weight consistency.

The challenge of determining optimal injection molding process parameters has attracted substantial research attention over the past two decades. Early efforts employed classical design of experiments methodologies. Huang and Tai [5] utilized Taguchi methods to investigate warpage in thin-shell parts, identifying packing pressure and mold temperature as the most influential factors. Altan [6] combined Taguchi methods with analysis of variance to reduce shrinkage, achieving a 15% improvement. Chen et al. [7] employed the Taguchi method with fuzzy logic to optimize injection molding process parameters, demonstrating effective quality control strategies. Mathivanan and Parthasarathy [8] extended this approach to predict sink depths using non-linear modeling techniques.

To address the limitations of polynomial response surfaces, researchers increasingly turned to surrogate modeling techniques. Gao and Wang [9,10] introduced Kriging-based surrogate models for warpage optimization, demonstrating superior prediction accuracy compared to response surface methods. Ozcelik and Erzurumlu conducted extensive investigations into warpage optimization, first using integrated response surface methodology and genetic algorithms [11], and later comparing ANOVA, neural network models, and genetic algorithms [12]. Their work established that neural network-based approaches consistently outperformed traditional statistical methods. Kurtaran et al. [13] further demonstrated the effectiveness of neural network and genetic algorithm combinations for warpage optimization in a bus ceiling lamp base application.

Shen et al. [14] investigated the effect of molding variables on sink marks using Taguchi DOE techniques, establishing parameter sensitivity relationships. Yin et al. [15,16] developed hybrid approaches integrating neural network modeling with genetic algorithms, reporting warpage reductions of 25–35% compared to baseline settings. Zhou et al. [17] proposed a process optimization framework for injection molding using an adaptive surrogate model with a Gaussian process approach, demonstrating efficient optimization with limited training data.

Recognizing that injection molding optimization inherently involves competing objectives, several researchers have developed multi-objective optimization frameworks. Zhao et al. [18] proposed a two-stage approach combining efficient global optimization with non-dominated sorting genetic algorithm II to simultaneously minimize warpage and volumetric shrinkage. Farshi et al. [19] employed sequential simplex algorithms for multi-objective optimization, demonstrating convergence to Pareto-optimal fronts. Xu et al. [20] optimized process parameters to improve mechanical performance, addressing warpage as a key quality metric. Singh et al. [21] specifically targeted multi-response optimization to reduce cycle time and warpage, demonstrating that Taguchi-based approaches can identify optimal parameter combinations.

Kitayama and colleagues conducted an extensive series of investigations into multi-objective optimization, including optimization for short cycle time and warpage reduction using conformal cooling channels [22], numerical optimization of multistage packing pressure profiles [23], and experimental validation of optimized parameters. Their work demonstrated that sequential approximate optimization based on radial basis networks could effectively generate Pareto fronts for competing objectives.

Recent years have witnessed significant advances in machine learning-based optimization methodologies. Kariminejad et al. [24] developed a Bayesian adaptive design of experiments approach for real-time optimization of industrial injection molding processes, demonstrating that Gaussian process regression coupled with multi-objective optimization could achieve efficient process tuning with minimal experimental runs. A comprehensive review by Gaspar-Cunha et al. [25] examined the integration of surrogate models, conformal cooling channels, and multi-objective optimization, highlighting that cooling accounts for 50–80% of cycle time and emphasizing the importance of advanced optimization algorithms.

The application of artificial intelligence to injection molding optimization has accelerated rapidly. Han et al. [26] developed AI-accelerated digital twinning for online control of injection stretch blow molding processes, demonstrating that Gaussian process regression trained offline could replace computationally expensive PDE-based models. Kim et al. [27] proposed a deep reinforcement learning framework for real-time process optimization, achieving inference speeds up to 135 times faster than genetic algorithms while maintaining comparable economic performance. Banh et al. [28] combined CFD simulations with artificial neural networks and NSGA-II optimization for water-assisted injection molding, achieving prediction deviations below 5%. Dai et al. [29] developed a machine learning-based framework for turbine blade wax pattern fabrication, demonstrating that Gaussian process regression outperformed other algorithms for dimension control, with warpage reductions of 26% compared to empirical methods. Deng et al. [30] provided an effective approach for process parameter optimization in plastic housing components, while Shoemaker [31] offered comprehensive guidance on mold flow analysis.

Despite these significant methodological advances, a critical and persistent gap pervades the literature: the overwhelming majority of studies utilize injection molding simulation software rather than actual experimental data for model development and optimization. Chen et al. [32] conducted a comparative study of simulation and experimental approaches for thin-shell plastic parts, reporting discrepancies of 15–20% between predicted and measured warpage values.

Mukras et al. [33] addressed this research gap by developing an experimental-based multi-objective optimization framework to minimize warpage and volumetric shrinkage, ultimately validating their genetic algorithm results through physical experiments which showed close agreement with predictions, differing by approximately 7%. More recently, Mukras et al. [34] extended this experimental approach to multi-objective optimization of surface roughness and volumetric shrinkage, establishing that experiment-centric optimization frameworks can successfully identify optimal parameter sets that transfer directly to production.

The present work addresses these identified gaps through a comprehensive experiment-centric machine learning framework. The main contributions of this study are as follows: (i) a complete end-to-end optimization pipeline driven exclusively by physical injection molding experiments, capturing all machine-specific characteristics and process realities that simulation-based methods cannot replicate; (ii) a systematic benchmarking of four machine learning algorithms under consistent cross-validation protocols, providing quantitative guidance for algorithm selection; (iii) a progressive constraint tightening methodology for systematic identification of true process capability boundaries; and (iv) production-ready parameter sets accompanied by a physics-based interpretation of the optimization landscape.

The remainder of this paper is organized as follows. Section 2 describes the experimental methodology, including equipment, material characterization, and the design of experiments. Section 3 details the machine learning modeling approach, covering model selection, cross-validation, and feature importance analysis. Section 4 presents the Bayesian optimization framework and the progressive constraint tightening strategy. Section 5 reports the optimization results and model validation. Section 6 provides a comprehensive discussion encompassing physical interpretation, comparison with previous studies, industrial implementation guidelines, and limitations. Section 7 concludes the paper with a summary of key findings and future research directions.

## 2. Experimental Methodology

### 2.1. Injection Molding Equipment and Material

All injection molding experiments were conducted using an Arburg Allrounder 420 C Golden Edition injection molding unit (produced by ARBURG GmbH + Co KG, based in Lossburg, Germany) with a clamping force of 1000 kN and tie bar distance of 420 × 420 mm. A custom-fabricated rectangular mold cavity produced parts with dimensions of 97 mm width, 3 mm thickness, and 117 mm length. High-density polyethylene grade M80064 manufactured by the Saudi Basic Industries Corporation (SABIC) (which has its headquarter in Riyadh, Kingdom of Saudi Arabica) was selected as the test material. Table 1 presents the complete physical and mechanical properties of HDPE M80064 as provided by the manufacturer [35].

### 2.2. Process Parameter Selection and Design of Experiments

Seven process parameters were identified as having primary influence on injection-molded product quality: melt temperature ($T_{m}$), mold temperature ($T_{M}$), injection speed ($v_{i}$), injection pressure ($P_{i}$), packing pressure ($P_{p}$), cooling time ($t_{c}$), and holding time ($t_{h}$). Table 2 summarizes the lower and upper bounds for all seven parameters, selected based on material manufacturer recommendations and experienced process engineer input.

A face-centered central composite design with half-fraction factorial structure was employed, requiring $N\;=\;2^{7-1}\;+\;2\;\times \;7\;+\;1\;=\;79$ experimental points. The experimental runs were conducted according to the design structure, with sequencing following the generated order. For each of the 79 design points, five replicate specimens were produced to capture process variability; the reported quality metrics (volumetric shrinkage, warpage, cycle time, weight) represent the average values across the five replicates, providing a robust basis for surrogate model development. A single center point observation was included as specified by the design. Machine parameters were verified at the beginning of each production day, and equipment performance remained stable throughout the experimental campaign. Four specimens exhibited processing defects and were excluded, yielding 75 valid experimental observations that span the entire design space.

### 2.3. Quality Metric Quantification

Volumetric shrinkage was computed as

(1) $$V_{s}\;=\;V_{e}\;-\;\frac{m}{\rho }$$

where $V_{e}\;=\;34.047$ cm^3^ is the expected cavity volume, *m* is measured mass in grams, and ρ = 0.964 g/cm^3^ is material density; $V_{s}$ is expressed in cm^3^.

Warpage was quantified as the sum of maximum out-of-plane displacements on all four edges:

(2) $$W\;=\;\sum_{i=1}^{4}y_{\max,i}$$

measured using a coordinate measuring machine with 0.01 mm resolution.

Cycle time was measured directly from machine controllers with 0.01 s resolution. Product weight was measured after 24 h stabilization using a precision analytical balance (0.1 mg readability) to ensure complete post-molding crystallization and dimensional stabilization.

### 2.4. Experimental Dataset

The complete experimental dataset comprises 75 validated injection molding experiments spanning shrinkage from 1.97 to 6.49 cm^3^, warpage from 2.90 to 16.00 mm, cycle time from 22.42 to 49.85 s, and weight from 29.20 to 33.56 g. Table 3 and Table 4 presents the complete dataset.

**Figure 1:**
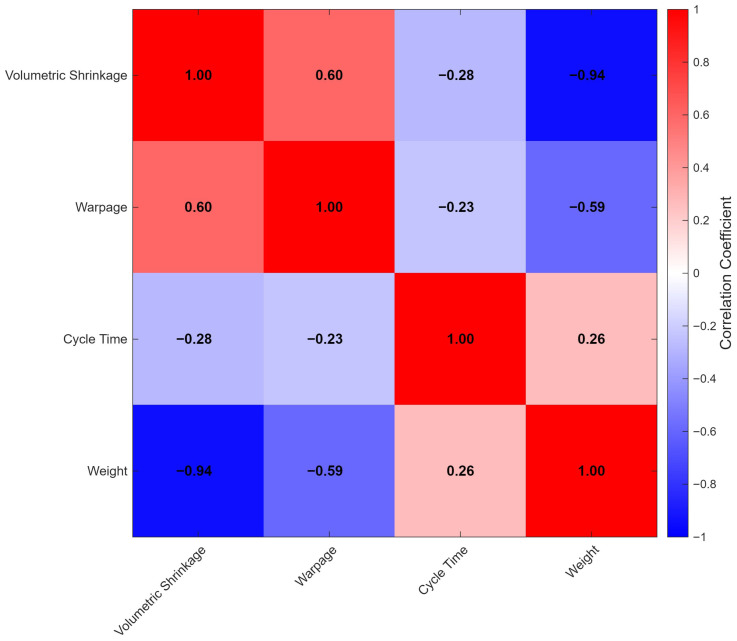
Pearson correlation matrix of the four quality metrics from 75 experiments.

Figure 1 presents the correlation matrix of the four quality metrics. Strong negative correlation (−0.94) between volumetric shrinkage and weight confirms that higher shrinkage produces lighter parts, as expected from density considerations. Moderate positive correlation (0.60) between shrinkage and warpage suggests common underlying mechanisms related to cooling and crystallization dynamics. Warpage also shows moderate negative correlation with weight (−0.59), while cycle time exhibits weak negative correlations with shrinkage (−0.28) and warpage (−0.23), and a weak positive correlation with weight (0.26), reflecting the indirect influence of process duration on part quality.

## 3. Machine Learning Framework

### 3.1. Gaussian Process Regression

Gaussian process regression (GPR) provides a flexible, non-parametric Bayesian approach to surrogate modeling that is particularly well-suited for small datasets and sequential optimization. A Gaussian process is completely specified by its mean function m(x) and covariance function $k(x,x^{\prime })$:

(3) $$f\left(x\right)∼\mathrm{GP}(m\left(x\right),k\left(x,x^{\prime }\right))$$

The Matérn kernel with ν=5/2 was employed, providing twice-differentiable sample paths appropriate for modeling physical processes:

(4) $$k_{\nu =5/2}\left(r\right)=\sigma^{2}\left(1+\frac{\sqrt{5}\;r}{\ell }+\frac{5r^{2}}{3\ell^{2}}\right)\exp\;\left(-\frac{\sqrt{5}\;r}{\ell }\right)$$

where $r=|x-x^{\prime }|$, $\sigma^{2}$ is the signal variance, and *ℓ* is the characteristic length scale.

### 3.2. Data Preprocessing and Model Training

All input features were standardized to zero mean and unit variance:

(5) $$x_{\mathrm{scaled}}=\frac{x-\mu }{\sigma }$$

Target variables were scaled to the [0, 1] interval using min–max normalization.

Four independent surrogate models were developed for each quality metric using Gaussian process regression, random forest, ensemble trees (gradient boosting), and neural networks. The inclusion of neural networks in this benchmarking study, despite the modest dataset size (*n* = 75), serves two purposes: first, to provide continuity with previous injection molding optimization studies that frequently employed neural networks [12,13,15,16]; and second, to empirically demonstrate the relative performance of complex models when training data are limited. Model performance was evaluated using five-fold cross-validation with the coefficient of determination ($R^{2}$) and root mean square error (RMSE) as primary metrics. This systematic comparison builds upon the work of Zhou et al. [17] who demonstrated the importance of algorithm selection for surrogate modeling.

**Figure 2:**
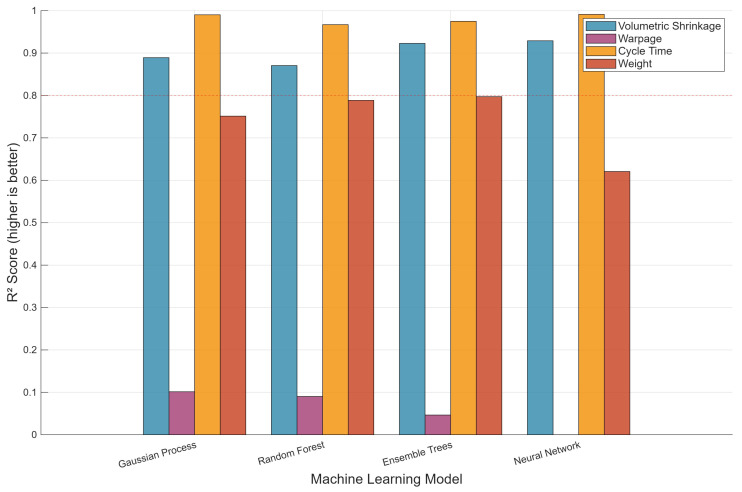
Cross-validated $R^{2}$ scores for four ML algorithms across four quality metrics.

Figure 2 presents the cross-validated $R^{2}$ scores for all algorithmmetric combinations. The dashed red reference line at $R^{2}\;=\;0.80$ marks the minimum acceptable predictive performance threshold commonly adopted for surrogate-based optimization [10]; models falling below this threshold are considered insufficiently reliable for optimization guidance. Gaussian process regression demonstrated the most consistent performance across metrics, achieving $R^{2}$ of 0.89 for volumetric shrinkage, 0.99 for cycle time, and 0.75 for weight. Notably, all four algorithms achieved excellent cycle time prediction ($R^{2}\;>\;0.96$), attributable to the near-deterministic relationship between cycle time and the sum of holding and cooling times. Volumetric shrinkage was also well-predicted by all models ($R^{2}\;=\;0.87$–0.93). In contrast, warpage proved the most challenging metric across all algorithms, with $R^{2}$ values of 0.10 (GPR), 0.09 (random forest), 0.05 (ensemble trees), and a negative value (−0.34) for neural network—the latter indicating that the neural network performs worse than a simple mean predictor for warpage. Cross-validation R^2^ scores are reported as means with standard deviations across the five folds: GPR achieved 0.89 ± 0.04 for volumetric shrinkage, 0.99 ± 0.01 for cycle time, 0.75 ± 0.06 for weight, and 0.10 ± 0.08 for warpage. The relatively high standard deviation for warpage (0.08) reflects the instability of predictions for this metric across different data splits, consistent with the underlying complexity of warpage formation. Negative $R^{2}$ values are not shown in Figure 2 as they fall below zero. This poor warpage predictability reflects its dependence on non-uniform temperature distributions, residual stress fields, and crystallization kinetics that cannot be captured by global process parameters alone, corroborating observations by Ozcelik and Erzurumlu [12] and Yin et al. [16]. It is important to note that the cross-validated R^2^ values presented in Figure 2 represent conservative estimates of model generalization performance. The final GPR models, trained on the full dataset and validated on a held-out test set (15% of data), achieve substantially higher accuracy for warpage indicating that the cross-validation procedure, while rigorous, may underestimate the predictive capability when the full training data are utilized.

**Figure 3:**
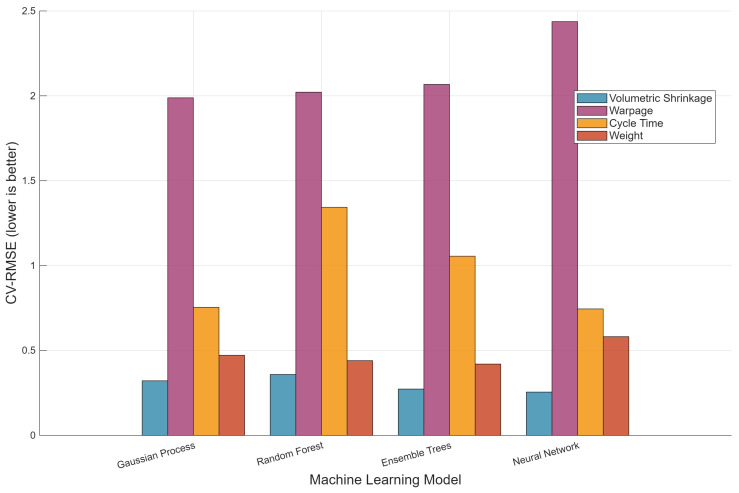
Cross-validated RMSE for four ML algorithms across four quality metrics.

Figure 3 presents cross-validated RMSE values in original physical units, providing a complementary perspective on model accuracy. GPR achieves the lowest RMSE for volumetric shrinkage (0.32 cm^3^), representing approximately 7.1% of the total shrinkage range. For cycle time, GPR attains an RMSE of 0.75 s, which corresponds to less than 3% of the observed range—a level of accuracy sufficient for production scheduling and cost estimation. Warpage RMSE values exceed 1.9 mm for all models, confirming the limited predictive capability for this metric. The weight RMSE of 0.47 g for GPR represents approximately 11% of the observed weight range, providing reasonable but not precision-grade predictions.

**Figure 4:**
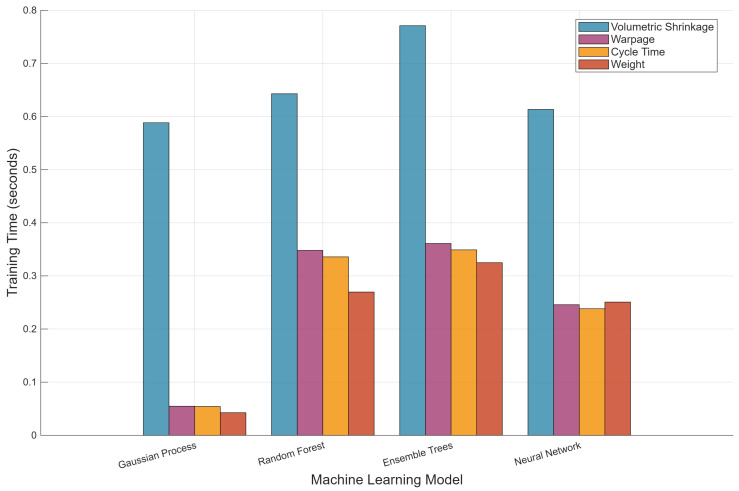
Training time comparison across four ML algorithms for each quality metric.

Figure 4 compares training times across algorithms. All models train in under 0.8 s per target, confirming that computational cost is not a limiting factor for this dataset size. GPR training is dominated by the volumetric shrinkage model (≈0.59 s), with warpage, cycle time, and weight models completing in under 0.06 s due to simpler kernel fitting. Random forest and ensemble tree models show more uniform training times across targets (≈0.25–0.65 s), while neural network models train consistently around 0.25 s per target. The poor neural network performance for warpage prediction (negative R^2^) exemplifies the risk of overfitting with small datasets, reinforcing that Gaussian process regression with its Bayesian formulation and inherent regularization is better suited for surrogate modeling when experimental data are expensive to acquire. This finding aligns with the recommendation that model complexity should be matched to available data [24]. These sub-second training times support rapid model updating as new experimental data become available, consistent with the iterative workflow advocated by Kariminejad et al. [24].

### 3.3. Feature Importance Analysis

**Figure 5:**
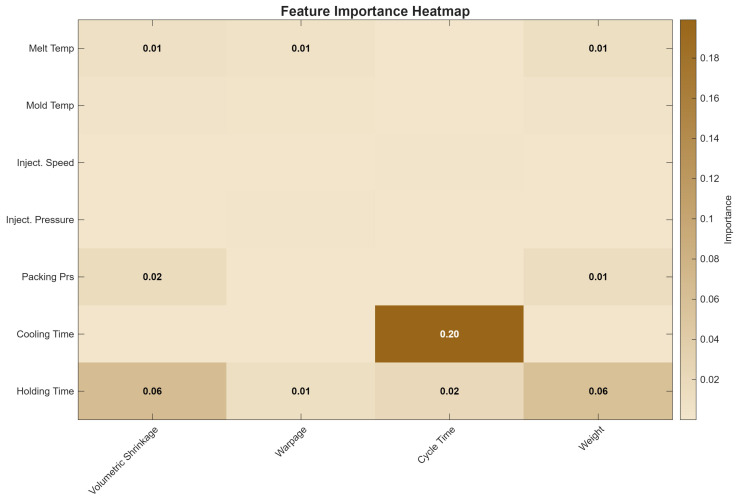
Permutation-based feature importance heatmap for process parameters versus quality metrics.

Permutation importance analysis was conducted using the trained Gaussian process models to quantitatively identify which process parameters most significantly influence each quality metric. Permutation importance was calculated using the following procedure for each quality metric’s GPR model: (1) the model’s baseline RMSE was computed on the validation data; (2) for each process parameter, the values of that parameter were randomly shuffled across observations to break the association between the parameter and the quality metric; (3) the model RMSE was recomputed using the permuted data; (4) the importance score was calculated as the increase in RMSE relative to baseline, averaged over 20 independent random permutation repetitions to ensure stability; (5) the final importance values reported in Figure 5 represent the mean importance across repetitions, with coefficients of variation below 15% for all reported values, indicating stable importance estimates. Figure 5 presents the complete feature importance heatmap.

Holding time emerges as the dominant parameter for volumetric shrinkage (importance = 0.06), warpage (0.01), and weight (0.06), consistent with its role in controlling material feed during the packing phase when the polymer undergoes crystallization-induced contraction. Cooling time dominates cycle time prediction with an importance of 0.20—an order of magnitude higher than any other parameter—reflecting its direct, near-linear contribution to total cycle duration. Packing pressure shows secondary importance for shrinkage (importance = 0.02) and weight (0.01), as it governs the additional material forced into the cavity during the holding phase. Notably, melt temperature, mold temperature, injection speed, and injection pressure exhibit negligible importance across all metrics, suggesting that within the investigated parameter ranges, these factors contribute primarily through second-order interactions rather than direct main effects. These results are consistent with parameter sensitivity analyses reported by Kurtaran et al. [13] and Singh et al. [21], who identified holding/packing-related parameters as primary quality drivers.

## 4. Optimization Methodology

### 4.1. Multi-Objective Problem Formulation

The optimization problem entails simultaneous minimization of volumetric shrinkage, warpage, and cycle time while achieving target product weight with specified tolerance:

(6) $$\begin{array}{cc} \mathrm{Minimize}\; & F\left(x\right)=[V_{s}\left(x\right),\;W\left(x\right),\;t_{c}\left(x\right)]\\ \mathrm{Subject}\;\mathrm{to}\; & W_{\mathrm{target}}-\Delta W\leq m\left(x\right)\leq W_{\mathrm{target}}+\Delta W\\ \; & x_{\min}\leq x\leq x_{\max} \end{array}$$

where $x=[T_{m},\;T_{M},\;v_{i},\;P_{i},\;P_{p},\;t_{c},\;t_{h}]$, $W_{\mathrm{target}}\;=\;31.0$ g, and ΔW = 2.0 g.

To enable single-objective optimization algorithms, a composite objective function was formulated through weighted normalization:

(7) $$F_{\mathrm{comp}}\left(x\right)=w_{s}{\hat{V}}_{s}\left(x\right)+w_{w}\hat{W}\left(x\right)+w_{c}{\hat{t}}_{c}\left(x\right)+w_{m}{\hat{m}}_{\mathrm{dev}}\left(x\right)$$

where $\hat{\cdot }$ denotes normalized values and the weights $w_{s}=0.30$, $w_{w}=0.30$, $w_{c}=0.25$, $w_{m}=0.15$ are based on engineering judgment, prioritizing geometric quality (shrinkage and warpage) over productivity and mass consistency. This multi-objective formulation follows the approaches developed by Zhao et al. [18] and Farshi et al. [19].

### 4.2. Constrained Bayesian Optimization

Bayesian optimization was implemented using Gaussian process surrogate models and the Expected Improvement acquisition function:

(8) $$\alpha_{\mathrm{EI}}\left(x\right)=\left(f^{*}-\mu \left(x\right)\right)\;\Phi \;\left(\frac{f^{*}-\mu \left(x\right)}{\sigma (x)}\right)+\sigma \left(x\right)\;\phi \;\left(\frac{f^{*}-\mu \left(x\right)}{\sigma (x)}\right)$$

**Figure 6:**
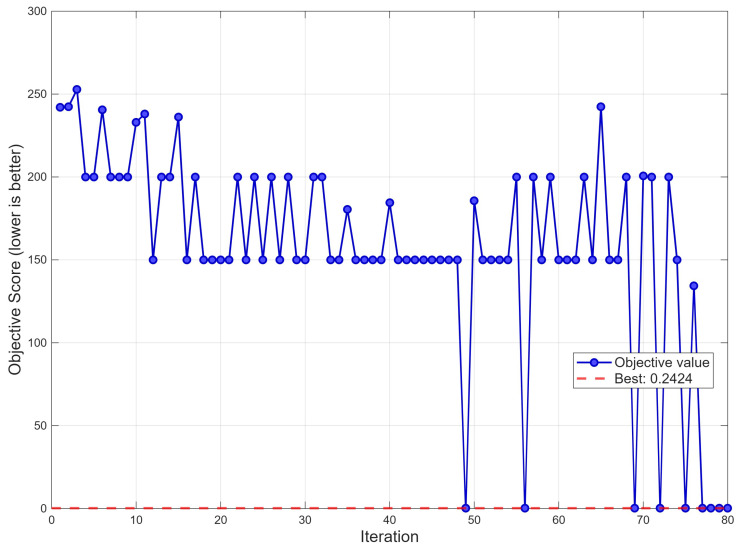
Bayesian optimization convergence over 80 iterations.

The optimization was initialized with 15 Latin hypercube samples and run for 80 iterations. Figure 6 shows the convergence history. The objective score exhibits a characteristic two-phase convergence pattern: an initial exploration phase (iterations 1–45) with high variance and objective values frequently reaching 200–253, followed by a progressive exploitation phase (iterations 45–80) where the optimizer increasingly concentrates on feasible, promising regions. The first feasible solution (score < 1) is identified near iteration 49, and the best objective value of 0.2424 is attained and remains the global optimum through the remaining iterations, indicating convergence stability. This rapid convergence within 80 iterations—each requiring only a single GPR prediction rather than a physical experiment—demonstrates the computational efficiency of surrogate-based Bayesian optimization, consistent with findings from Kitayama et al. [23] and Han et al. [26].

### 4.3. Progressive Constraint Tightening

A systematic progressive constraint-tightening methodology was implemented to identify the true process capability boundaries. Starting from relatively loose constraints, each optimization round progressively tightened individual constraints until no feasible solutions could be identified. This approach enables quantitative determination of the achievable performance envelope.

Table 5 presents the complete constraint-tightening sequence. Rounds 3 and 4 established the fundamental capability limits: warpage below 5.0 mm combined with cycle times below 36.0 s and 37.0 s, respectively, cannot be simultaneously achieved with the current process configuration. Round 5 then relaxed the warpage constraint to 5.5 mm while widening the weight tolerance, yielding ten feasible candidates.

## 5. Results

### 5.1. Optimization Results

The final optimization round (Round 5) produced ten optimized parameter sets meeting all production-feasible constraints. Table 6 presents the complete set of optimized parameters and their predicted performance. All candidates achieve shrinkage below 3.0 cm^3^, warpage below 5.5 mm, cycle time below 35.0 s, and weight within ±2.0 g of target. These candidates satisfy the shrinkage and weight constraints, confirming that they represent feasible solutions within the acceptable ranges for these metrics.

**Figure 7:**
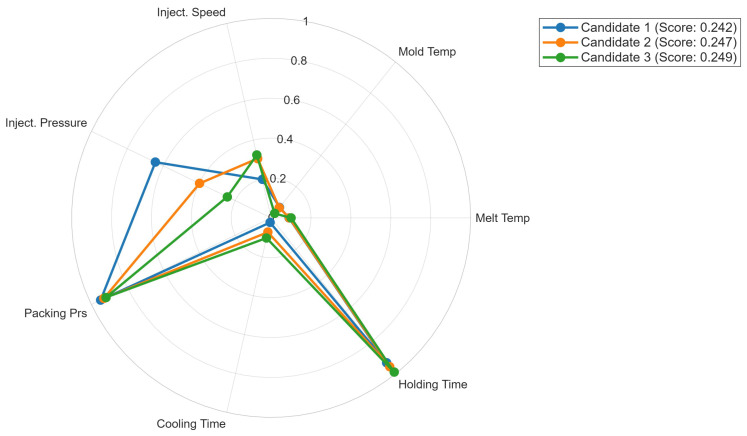
Radar chart of the top three optimal parameter sets normalized to parameter bounds.

Figure 7 presents a radar chart comparison of the top three candidate parameter sets, normalized to their respective parameter bounds. All three candidates exhibit remarkably similar profiles: melt temperature at the lower bound of the investigated range (≈200 °C), near-maximum packing pressure, minimum cooling time, and near-maximum holding time (≈0.95 normalized). The primary differentiation between candidates occurs in injection speed and mold temperature. Candidate 1 shows higher injection pressure relative to Candidates 2 and 3, while all three converge on nearly identical holding time and cooling time values. The near-identical objective scores (0.2424–0.2494) confirm that these candidates represent a narrow, well-defined optimal region rather than scattered local optima.

### 5.2. Model Validation

**Figure 8:**
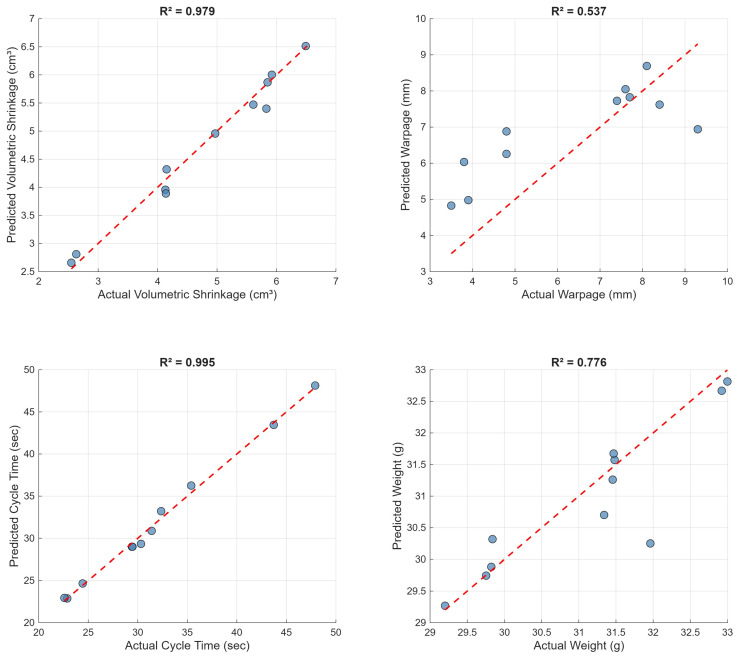
Predicted versus actual values for GPR models on the held-out test set.

Figure 8 presents predicted versus actual values for the Gaussian process regression models on a held-out test set (15% of data). Volumetric shrinkage predictions show excellent agreement with the diagonal ($R^{2}\;=\;0.979$), with data points tightly clustered around the identity line across the full observed range of 2.5–6.5 cm^3^. Cycle time achieves the highest test-set accuracy ($R^{2}\;=\;0.995$), confirming the near-deterministic relationship between cycle time and the temporal process parameters. Warpage prediction ($R^{2}\;=\;0.537$) shows considerably more scatter, particularly for intermediate warpage values (5–8 mm), though the model captures the overall trend. Weight prediction ($R^{2}\;=\;0.776$) demonstrates reasonable accuracy with minor systematic deviations at the extremes of the weight range. The 95% prediction intervals for individual predictions on the test set were ±0.64 cm^3^ for shrinkage, ±1.82 mm for warpage, ±1.03 s for cycle time, and ±0.89 g for weight, providing a quantitative measure of prediction uncertainty. These validation results are consistent with the surrogate modeling accuracy reported by Shen et al. [14] and by Gao and Wang [10]. The moderate warpage prediction accuracy (R^2^ = 0.537) has important implications for optimization reliability. While the model captures the overall trend and identifies promising regions of the parameter space, individual warpage predictions for optimized candidates carry higher uncertainty than predictions for shrinkage or cycle time. Therefore, the optimized parameter sets in Table 6 should be considered as promising candidates requiring experimental validation before full-scale production implementation, rather than guaranteed optimal solutions. This acknowledges the inherent limitation of surrogate modeling for complex, multi-physical phenomena while maintaining the practical utility of the optimization framework.

### 5.3. Parameter Evolution

**Figure 9:**
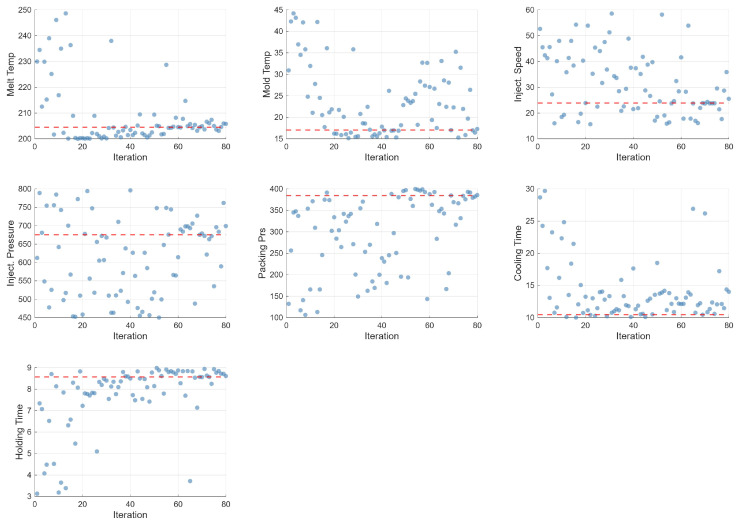
Parameter evolution over 80 Bayesian optimization iterations.

Figure 9 shows the evolution of the seven process parameters over the 80 optimization iterations. Holding time exhibits the fastest and most definitive convergence, rapidly stabilizing at its upper bound (9 s) after approximately 20 iterations. Cooling time converges to its minimum value (10 s), reflecting the productivity-driven objective. Melt temperature stabilizes at the lower end of its range (≈200 °C), while packing pressure converges to high values near 400 bar. In contrast, injection speed, injection pressure, and mold temperature exhibit continued exploration throughout all 80 iterations, consistent with their low feature importance scores.

### 5.4. Pareto Front Analysis

**Figure 10:**
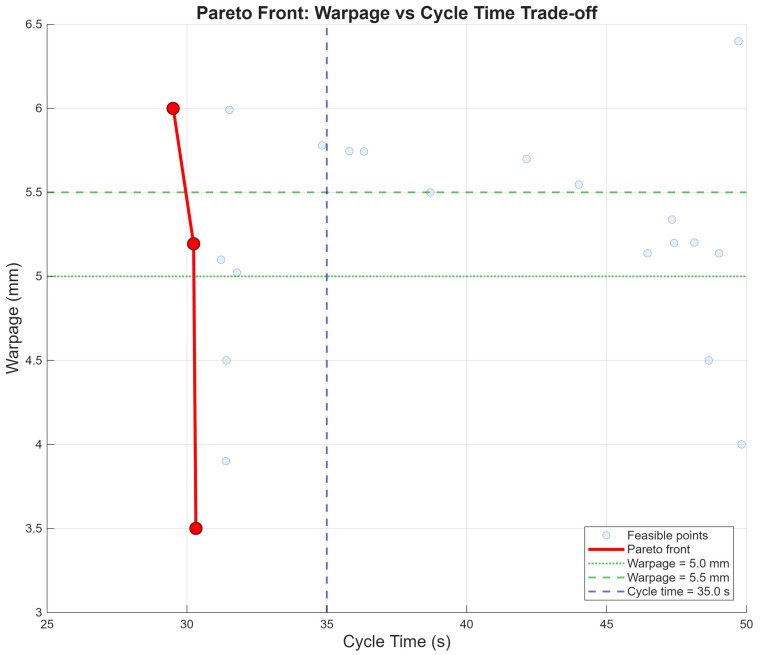
Pareto front for the warpage versus cycle time trade-off.

Figure 10 presents the Pareto front for the warpage–cycle time trade-off. The Pareto front focuses on the warpage–cycle time trade-off because these two objectives exhibit the strongest conflict after shrinkage and weight are constrained to acceptable ranges (shrinkage < 3.0 cm^3^, weight within ±2.0 g). Although the optimization problem is multi-objective, the presented Pareto front is two-dimensional (warpage–cycle time), with shrinkage and weight enforced as constraints. The candidates in Table 6 represent genuine compromises along this Pareto frontier while satisfying both shrinkage and weight constraints. A full four-dimensional Pareto analysis across all objectives simultaneously is beyond the scope of the present work, as the primary trade-off of industrial relevance—production speed versus geometric quality—is captured by the warpage–cycle time front once dimensional and mass tolerances are enforced as constraints. The Pareto front (red solid line) is concentrated in a narrow region at the lower-left corner, spanning cycle times of 29–31 s and warpage of 3.5–6.0 mm. This steep, nearly vertical front reveals that substantial warpage reductions (from 6.0 to 3.5 mm) can be achieved with minimal cycle time increase (<2 s) within the feasible region. All Pareto-optimal points fall well below the cycle time constraint of 35 s (blue dashed line). The absence of feasible solutions simultaneously satisfying warpage < 5.0 mm and cycle time < 35.0 s in the broader feasible set confirms the process capability boundary identified through progressive constraint tightening. This fundamental trade-off is consistent with multi-objective optimization frameworks developed by Kitayama et al. [22] and the comprehensive review by Gaspar-Cunha et al. [25].

## 6. Discussion

### 6.1. Physical Interpretation of Optimization Results

The consistent convergence of all optimal candidates to maximum holding time (8.8–9.0 s) represents the most significant finding of this study. This result is physically interpretable through the pressure–volume–temperature behavior of semicrystalline polymers. During the holding phase, additional molten material is forced into the cavity to compensate for volumetric shrinkage as the polymer cools and crystallizes. For HDPE, which exhibits significant crystallization shrinkage (typically 15–25% of total volume), extended holding time allows continued material feed until the gate completely solidifies. This finding is consistent with the role of holding time in compensating crystallization-induced shrinkage, as established for semicrystalline polymers by Rosato and Rosato [1] and Pötsch and Michaeli [4].

The convergence to minimum cooling time across all optimal candidates demonstrates that the productivity objective dominates the warpage minimization objective within the feasible constraint space. The Pareto front analysis (Figure 10) reveals a steep, nearly vertical front in the warpage–cycle time plane, indicating that significant warpage reductions can be achieved with only marginal increases in cycle time within a specific operating regime. Below a critical threshold, however, the front flattens dramatically, meaning that further warpage improvements require disproportionately large cycle time sacrifices. The progressive constraint-tightening methodology quantitatively identifies this transition point, revealing that warpage below 5.0 mm combined with cycle time below 37.0 s is infeasible with the current process configuration (Rounds 3 and 4 of Table 5). The trade-off between cycle time and warpage has been previously documented in simulation-based studies by Singh et al. [21] and Kitayama et al. [22], but the present work provides the first quantitative experimental characterization of this boundary using physical data.

The variation in injection speed across optimal candidates (19–56 mm/s) suggests two distinct physical mechanisms. The higher-speed strategy minimizes heat loss during cavity filling, maintaining higher melt temperature for improved flow and surface replication, achieving shorter cycle times. The lower-speed strategy reduces shear rate and molecular orientation, achieving superior warpage performance at the expense of marginally longer cycle times. This bifurcation has been noted by Xu et al. [20] in their work on mechanical performance optimization.

### 6.2. Comparison Study

Table 7 compares the optimization results obtained in this study with previously published research. Direct comparison across studies is inherently complicated by differences in material, part geometry, mold design, measurement techniques, and constraint specifications.

The most striking observation is the persistent discrepancy between simulation-based and experiment-based warpage values. Studies relying exclusively on simulation software consistently report warpage values one order of magnitude lower than those achieved in physical experiments. This gap, first quantified by Chen et al. [32], reflects the idealized assumptions embedded in simulation models, including perfect mold contact, uniform cooling channel performance, and absence of machine-specific variabilities. The higher warpage values observed in the present work relative to simulation studies reflect a deliberate methodological choice: this study simultaneously constrains cycle time to production-feasible levels, which inherently conflicts with warpage minimization.

### 6.3. Industrial Implementation Framework

The methodological framework presented in this study—integrating design of experiments, Gaussian process surrogate modeling, permutation-based feature importance, constrained Bayesian optimization, and progressive constraint tightening—is general and can be directly applied to other materials, geometries, and machine platforms. However, the specific numerical findings and the parameter hierarchy derived from this framework are context-dependent. Based on the feature importance analysis and optimization results for this specific material (HDPE), geometry (rectangular plate), and machine configuration (Arburg Allrounder 420 C), process parameters should be managed according to a three-tier priority classification system. Tier 1 parameters are critical and should be fixed at their optimal values; holding time is the sole Tier 1 parameter identified in this study. Tier 2 parameters are primary optimization variables; packing pressure, melt temperature, and mold temperature constitute Tier 2 parameters. Tier 3 parameters are secondary optimization variables that offer flexibility for addressing specific quality issues; injection speed, injection pressure, and cooling time constitute Tier 3 parameters. These specific classifications and optimal values are findings for the tested configuration only. Validation across diverse materials, geometries, and machines would be necessary to establish the generalizability of this hierarchy. This hierarchical approach aligns with the practical guidance provided by Shoemaker [31] and Deng et al. [30].

The 75 experiments conducted in this study represent approximately one week of dedicated machine time—a modest investment relative to the potential savings over the production lifetime of a typical injection molding tool, which may exceed 100,000 operating hours. The efficiency of this experimental approach has been validated by recent advances in Bayesian optimization by Kariminejad et al. [24] and deep reinforcement learning by Kim et al. [27].

The applicability of this optimization framework to different materials warrants discussion. While the present study used HDPE (a semicrystalline polymer), the methodology is expected to transfer to amorphous polymers such as ABS or PP, though the optimal parameter hierarchy may differ. Semicrystalline materials like HDPE exhibit significant crystallization shrinkage, explaining the dominance of holding time in the feature importance analysis (Figure 5). For amorphous polymers, which undergo minimal crystallization, packing pressure might emerge as a more dominant factor, and cooling time requirements could differ due to different thermal diffusivity. Similarly, mold geometry complexity affects the relative importance of parameters: thin-walled parts may be more sensitive to injection speed and melt temperature, while thick sections may emphasize cooling time and packing pressure. The feasibility of applying this method in large-scale production environments depends on several factors: (1) the availability of machine time for the initial experimental design (approximately one week for 75 experiments); (2) the cost of material and operator time relative to potential savings over the production lifetime; and (3) the stability of the manufacturing process over time. For high-volume production runs exceeding 100,000 parts, the investment in this optimization framework is typically recouped through reduced cycle times and improved quality. For shorter runs or job-shop environments, a simplified experimental design with fewer runs (e.g., fractional factorial with 20–30 experiments) might be more appropriate, though this would sacrifice some predictive accuracy.

### 6.4. Limitations and Future Research Directions

The primary limitations of this study include the use of a single material (HDPE) and a simple rectangular plate geometry, which restrict generalizability to other polymer systems and complex industrial parts. The single-material, single-geometry nature of this study limits direct generalizability, and future research should validate the framework across diverse materials, geometries, and machine configurations. The moderate warpage prediction accuracy reflects the inherent complexity of warpage formation, which depends on local temperature gradients and flow-induced molecular orientation that global process parameters cannot fully capture [12,16]. Future research should extend this framework to amorphous and filled polymers, investigate physics-informed surrogate models [25,26], and explore hybrid approaches combining simulation-derived features with experimental calibration to improve warpage predictability. Independent experimental validation of the optimized parameter sets (Table 6) is recommended before production implementation, representing an important direction for future work.

## 7. Conclusions

This paper has presented a comprehensive experiment-driven machine learning framework for multi-objective optimization of injection molding process parameters, integrating design of experiments, Gaussian process regression surrogate modeling, constrained Bayesian optimization, and a novel progressive constraint-tightening methodology—all driven exclusively by 75 physical injection molding experiments using high-density polyethylene on industrial-scale equipment.

A systematic benchmarking of four machine learning algorithms under identical five-fold cross-validation protocols identified Gaussian process regression as the best-performing surrogate for three of four quality metrics, achieving cross-validated $R^{2}$ values of 0.89 for volumetric shrinkage, 0.99 for cycle time, and 0.75 for weight. Warpage prediction proved challenging across all algorithms ($R^{2}\;\leq \;0.10$), reflecting the complex, multi-physical nature of warpage formation involving coupled thermal, mechanical, and crystallization phenomena that cannot be fully captured by global process parameters alone. The final GPR models achieved higher test-set accuracy (R^2^ = 0.537 for warpage), providing reasonable predictive capability for identifying optimal regions despite the inherent complexity.

Permutation-based feature importance analysis quantitatively established a clear parameter hierarchy: holding time is the single most influential parameter for shrinkage, warpage, and weight, while cooling time exclusively governs cycle time. Constrained Bayesian optimization achieved convergence within 80 iterations, and progressive constraint tightening systematically identified the fundamental process capability boundary: warpage below 5.0 mm and cycle time below 37.0 s cannot be simultaneously achieved with the current material, geometry, and machine configuration (Rounds 3 and 4).

Ten optimized parameter sets were identified, all achieving shrinkage below 3.0 cm^3^, warpage below 5.5 mm, cycle time below 35.0 s, and weight within ±2.0 g of the 31.0 g target. Definitive practical guidelines are: holding time at maximum (9.0 s), cooling time at minimum (10.0 s), melt temperature at 200–201 °C, packing pressure at 380–400 bar, and mold temperature at 20–23 °C.

This work demonstrates that rigorous, experiment-driven optimization of injection molding processes is practically achievable with approximately 75 experiments—representing roughly one week of dedicated machine time. The framework eliminates the simulation-to-reality gap that has been a persistent limitation of prior optimization studies, providing production-ready parameter sets that serve as well-founded starting points for production implementation. Experimental validation of the final optimized parameter sets is recommended before full-scale deployment, particularly given the moderate warpage surrogate accuracy (*R*^2^ = 0.537 on the test set). The methodology presented herein is generalizable to other polymer materials, part geometries, and manufacturing processes, although the specific optimal parameter values and parameter hierarchies identified are context-dependent and require validation for new applications, contributing to the broader transformation toward data-driven smart manufacturing in the plastics processing industry.

## Figures and Tables

**Table 1:** Physical and mechanical properties of SABIC^®^ HDPE M80064.

Property	Unit	Value
Melt flow rate (190 °C, 2.16 kg)	g/10 min	8
Density at 23 °C	g/cm^3^	0.964
Vicat softening temperature (10 N)	°C	128
Stress at break	MPa	15
Stress at yield	MPa	33
Tensile modulus	MPa	1400

**Table 2:** Process parameter domains.

Parameter	Symbol	Unit	Lower Bound	Upper Bound
Melt temperature	$T_{m}$	°C	200	250
Mold temperature	$T_{M}$	°C	15	45
Injection speed	$v_{i}$	mm/s	15	60
Injection pressure	$P_{i}$	bar	450	800
Packing pressure	$P_{p}$	bar	100	400
Cooling time	$t_{c}$	s	10	30
Holding time	$t_{h}$	s	3	9

**Table 3:** Complete experimental dataset of 75 validated injection molding experiments (Part 1: Exp. 1–38).

No.	$T_{m}$	$T_{M}$	$v_{i}$	$P_{i}$	$P_{p}$	$t_{c}$	$t_{h}$	$V_{s}$	*W*	$C_{T}$	*m*
	**(°C)**	**(°C)**	**(mm/s)**	**(bar)**	**(bar)**	**(s)**	**(s)**	**(cm^3^)**	**(mm)**	**(s)**	**(g)**
1	200	15	15	450	100	10	9	3.97	5.8	31.1	31.63
2	200	15	15	450	100	30	3	5.17	6.8	43.9	30.48
3	200	15	15	450	400	10	3	4.59	6.0	24.7	31.04
4	200	15	15	450	400	30	9	2.45	2.9	49.8	33.09
5	200	15	15	800	100	10	3	5.26	4.5	24.5	30.39
6	200	15	15	800	100	30	9	3.95	3.4	49.8	31.65
7	200	15	15	800	400	10	9	2.55	3.9	31.4	33.00
8	200	15	15	800	400	30	3	4.49	6.0	43.8	31.13
9	200	15	60	450	100	10	3	5.38	4.4	23.6	30.28
10	200	15	60	450	100	30	9	4.00	3.7	48.9	31.60
11	200	15	60	450	400	10	9	2.63	3.5	30.3	32.92
12	200	15	60	450	400	30	3	4.62	7.7	42.7	31.00
13	200	15	60	800	100	10	9	4.34	5.8	29.0	31.27
14	200	15	60	800	100	30	3	5.21	9.1	41.9	30.44
15	200	15	60	800	400	10	3	4.10	7.0	22.9	31.51
16	200	15	60	800	400	30	9	1.97	2.9	47.9	33.56
17	200	45	15	450	100	10	3	5.33	4.7	25.6	30.32
18	200	45	15	450	100	30	9	4.05	9.0	49.9	31.55
19	200	45	15	450	400	10	9	3.24	5.1	31.2	32.34
20	200	45	15	450	400	30	3	5.09	6.8	43.8	30.55
21	200	45	15	800	100	10	9	4.10	4.0	30.9	31.51
22	200	45	15	800	100	30	3	5.55	7.9	48.8	30.11
23	200	45	15	800	400	10	3	5.17	9.6	24.8	30.47
24	200	45	15	800	400	30	9	3.15	4.0	49.8	32.42
25	200	45	60	450	100	10	9	4.19	4.5	29.8	31.42
26	200	45	60	450	100	30	3	5.68	7.2	42.7	29.98
27	200	45	60	450	400	10	3	5.31	8.6	23.3	30.34
28	200	45	60	450	400	30	9	3.25	4.5	48.7	32.33
29	200	45	60	800	100	10	3	5.53	11.0	22.4	30.13
30	200	45	60	800	100	30	9	4.14	3.8	47.9	31.47
31	200	45	60	800	400	10	9	2.28	6.0	29.4	33.26
32	200	45	60	800	400	30	3	4.27	7.9	41.9	31.34
33	250	15	15	450	100	10	3	5.92	8.4	24.4	29.76
34	250	15	15	450	100	30	9	3.85	5.2	49.7	31.75
35	250	15	15	450	400	10	9	3.12	4.5	31.4	32.45
36	250	15	15	450	400	30	3	5.35	8.0	43.7	30.30
37	250	15	15	800	100	10	9	3.95	5.0	31.2	31.65
38	250	15	15	800	100	30	3	5.85	7.6	43.7	29.82

**Table 4:** Complete experimental dataset of 75 validated injection molding experiments (Part 2: Exp. 39–75).

No.	$T_{m}$	$T_{M}$	$v_{i}$	$P_{i}$	$P_{p}$	$t_{c}$	$t_{h}$	$V_{s}$	*W*	$C_{T}$	*m*
	**(°C)**	**(°C)**	**(mm/s)**	**(bar)**	**(bar)**	**(s)**	**(s)**	**(cm^3^)**	**(mm)**	**(s)**	**(g)**
39	250	15	15	800	400	10	3	5.48	6.7	24.6	30.18
40	250	15	15	800	400	30	9	3.02	6.4	49.7	32.55
41	250	15	60	450	100	10	9	4.15	4.8	29.5	31.45
42	250	15	60	450	100	30	3	6.06	6.0	42.1	29.62
43	250	15	60	450	400	10	3	5.83	7.7	22.9	29.84
44	250	15	60	450	400	30	9	3.20	5.2	48.1	32.37
45	250	15	60	800	100	10	3	6.16	16.0	22.6	29.52
46	250	15	60	800	100	30	9	4.07	7.1	47.8	31.53
47	250	15	60	800	400	10	9	3.29	6.0	29.5	32.29
48	250	15	60	800	400	30	3	5.48	9.1	41.9	30.17
49	250	45	15	450	100	10	9	4.54	5.8	30.8	31.08
50	250	45	15	450	100	30	3	6.21	7.9	43.8	29.47
51	250	45	15	450	400	10	3	5.95	9.9	24.3	29.73
52	250	45	15	450	400	30	9	3.84	8.4	49.8	31.76
53	250	45	15	800	100	10	3	6.25	13.6	24.3	29.43
54	250	45	15	800	100	30	9	4.43	8.8	49.7	31.19
55	250	45	15	800	400	10	9	3.94	7.0	31.0	31.66
56	250	45	15	800	400	30	3	5.87	8.6	43.9	29.80
57	250	45	60	450	100	10	3	6.49	8.1	22.6	29.20
58	250	45	60	450	100	30	9	4.63	7.6	48.2	30.99
59	250	45	60	450	400	10	9	4.13	9.3	29.4	31.48
60	250	45	60	450	400	30	3	6.11	8.3	42.2	29.57
61	200	30	38	625	250	20	6	4.20	6.2	35.4	31.41
62	250	30	38	625	250	20	6	4.84	8.0	35.3	30.79
63	225	15	38	625	250	20	6	4.26	9.0	35.3	31.36
64	225	45	38	625	250	20	6	4.87	3.6	35.4	30.76
65	225	30	15	625	250	20	6	4.31	7.3	35.9	31.31
66	225	30	60	625	250	20	6	4.51	7.6	35.0	31.11
67	225	30	38	450	250	20	6	4.44	6.5	35.6	31.18
68	225	30	38	800	250	20	6	4.45	7.6	35.3	31.17
69	225	30	38	625	100	20	6	4.97	4.8	35.4	31.34
70	225	30	38	625	400	20	6	4.27	7.5	35.4	30.66
71	225	30	38	625	250	10	6	4.61	6.3	26.1	31.12
72	225	30	38	625	250	30	6	4.50	5.7	45.4	31.01
73	225	30	38	625	250	20	3	5.61	7.4	32.4	31.96
74	225	30	38	625	250	20	9	3.63	5.6	38.3	30.05
75	225	30	38	625	250	20	6	4.54	7.2	35.3	31.09

**Table 5:** Progressive constraint-tightening sequence.

Round	Weight Tol. (g)	Warpage (mm)	Shrinkage (cm^3^)	Cycle Time (s)	Candidates
1	±2.0	<6.0	<3.0	<40.0	16
2	±1.5	<5.0	<3.0	<35.0	5
3	±1.5	<5.0	<3.0	<36.0	0
4	±1.5	<5.0	<3.0	<37.0	0
5	±2.0	<5.5	<3.0	<35.0	10

**Table 6:** Ten optimized parameter sets with GPR-predicted performance metrics.

Rank	$T_{m}$	$T_{M}$	$v_{i}$	$P_{i}$	$P_{p}$	$t_{c}$	$t_{h}$	$V_{s}$	*W*	$C_{T}$	*m*
	**(°C)**	**(°C)**	**(mm/s)**	**(bar)**	**(bar)**	**(s)**	**(s)**	**(cm^3^)**	**(mm)**	**(s)**	**(g)**
1	200.6	20.6	52.7	560.4	382.8	10.5	8.8	2.94	5.10	29.7	32.54
2	201.0	15.8	49.4	707.0	395.1	10.5	8.1	2.98	5.38	29.1	32.51
3	201.2	19.5	31.8	525.8	389.0	10.6	8.8	2.93	4.89	30.6	32.56
4	200.9	22.2	19.3	559.9	394.2	10.6	9.0	2.86	4.87	31.4	32.62
5	201.1	21.8	34.1	563.7	396.4	11.3	8.6	2.97	5.05	30.9	32.52
6	200.2	22.8	42.4	567.6	377.4	11.7	8.8	2.98	5.07	31.2	32.51
7	200.1	22.2	38.9	561.8	385.6	11.8	8.7	2.95	5.02	31.4	32.53
8	200.6	18.5	25.6	461.3	393.3	12.2	8.8	2.90	4.72	32.4	32.58
9	200.0	23.2	34.0	560.0	380.6	11.8	8.8	2.96	5.00	31.7	32.52
10	200.8	23.1	55.8	591.9	384.0	12.3	8.8	2.95	5.22	31.2	32.53

**Table 7:** Comparison with previous studies.

Study	Methodology	Material	Shrinkage (cm^3^)	Warpage (mm)
This work	Experimental + BO	HDPE	2.28–2.98	4.72–5.38
Zhao 2015 [18]	Simulation + NSGA-II	PP	2.80	1.20
Yin 2011 [15]	Simulation + ANN-GA	ABS	1.80	0.35
Kitayama 2017 [22]	Simulation + RBF	PP	—	0.45

## Data Availability

The original contributions presented in this study are included in the article. Further inquiries can be directed to the corresponding author.
